# Oxidative changes in lipids, proteins, and antioxidants in yogurt during the shelf life

**DOI:** 10.1002/fsn3.493

**Published:** 2017-07-25

**Authors:** Anna Citta, Alessandra Folda, Valeria Scalcon, Guido Scutari, Alberto Bindoli, Marco Bellamio, Emiliano Feller, Maria Pia Rigobello

**Affiliations:** ^1^ Department of Biomedical Sciences University of Padova Padova Italy; ^2^ Institute of Neuroscience (CNR) Padova Italy; ^3^ Centrale del Latte di Vicenza s.p.a. Vicenza Italy

**Keywords:** antioxidants, lipid peroxidation, protein oxidation, shelf life, yogurt

## Abstract

Oxidation processes in milk and yogurt during the shelf life can result in an alteration of protein and lipid constituents. Therefore, the antioxidant properties of yogurt in standard conditions of preservation were evaluated. Total phenols, free radical scavenger activity, degree of lipid peroxidation, and protein oxidation were determined in plain and skim yogurts with or without fruit puree. After production, plain, skim, plain berries, and skim berries yogurts were compared during the shelf life up to 9 weeks. All types of yogurts revealed a basal antioxidant activity that was higher when a fruit puree was present but gradually decreased during the shelf life. However, after 5–8 weeks, antioxidant activity increased again. Both in plain and berries yogurts lipid peroxidation increased until the seventh week of shelf life and after decreased, whereas protein oxidation of all yogurts was similar either in the absence or presence of berries and increased during shelf life. During the shelf life, a different behavior between lipid and protein oxidation takes place and the presence of berries determines a protection only against lipid peroxidation.

## 1 INTRODUCTION

Free radicals and other reactive oxygen species (ROS) may cause to living and food systems an oxidative damage that, however, can be prevented by several types of antioxidants (Halliwell & Gutteridge, 2015; Gülçin, 2012). For instance, in the human diet, fruits are relevant components and provide nutrients such as carbohydrates, minerals, and vitamins together with phytochemicals which include polyphenols and carotenoids, all endowed with potent antioxidant properties (Jacob et al., 2012). In addition to the antioxidants of endogenous or exogenous origin, a contribution to the antioxidant defense is also given by proteins, peptides, and amino acids (Power, Jakeman, & Fitzgerald, 2013; Sarmadi & Ismail, 2010). For instance, the tripeptide glutathione is a well‐known antioxidant acting both directly and as a substrate of glutathione peroxidase which is able to convert hydrogen peroxide to water. The dipeptide carnosine (β‐alanyl‐L‐histidine), particularly abundant in skeletal muscle and brain, may act as a free radical scavenger and metal chelator (Decker, Livisay, & Zhou, 2000; Baye et al., 2016).

Milk and dairy products, basic foods for human nutrition, can also contribute to the body defense against oxidants. However, oxidation processes in milk can result in sharp off‐flavors and a decline of its nutritional properties. Consequently, the oxidative stability of milk and dairy products has great importance especially considering their shelf life. The antioxidant activity is due to the natural antioxidants present in milk (Lindmark‐Månsson & Akesson, 2000); and is in part depending from the food supply to herbivores. For instance, phenolic compounds, products of the secondary metabolism in plants, are effective natural antioxidants and are present in noticeable amounts in the ruminant milk, mostly deriving from the feeding (O'Connell & Fox, 2001). In addition, both fermented milk and yogurt contain bioactive peptides, formed by hydrolysis of milk proteins during the fermentation process, and also endowed with antioxidant activity (Power et al., 2013; Pihlanto, 2006; Aloğlu & Oner, 2001). However, there is still a lack of knowledge on the antioxidant capacity of dairy products such as probiotic yogurts containing different types of fruits. In this case, the antioxidant power largely depends on the presence of added fruit puree containing various bioactive compounds such as tocopherols, carotenoids, ascorbate, and especially phenolic compounds which are good contributors to the total antioxidant capacity of yogurt (O'Connell & Fox, 2001; Şengül, Erkaya, Şengül, & Yildiz, 2012).

This study aims to determine how the antioxidant power of yogurt changes during shelf life in standard conditions of preservation. Therefore, we examined the antioxidant capacity of a plain or skim yogurt with or without added fruit puree in relation to the total content of phenols and free radical scavenging capacity. Furthermore, lipoperoxidation processes and the content of carbonyl groups, an index of protein oxidation, were also evaluated, showing a divergent behavior.

## 2 EXPERIMENTAL

### 2.1 Materials

Trolox C, ABTS (2, 2′‐azinobis(3‐ethylbenzothiazoline 6‐sulfonate)) were purchased from Fluka‐Sigma‐Aldrich (St. Louis, MO, USA). DPPH (1,1‐diphenyl‐2‐picrylhydrazyl) and Folin‐Ciocalteau reagent were obtained from Sigma‐Aldrich (St. Louis, MO, USA). All types of yogurts analyzed in this study were obtained from Centrale del Latte di Vicenza (Vicenza, IT), *Lactobacillus delbrueckii subs. bulgaricus* and *Streptococcus thermophilus* from Danisco (Copenhagen, DK), and fruit puree from Zuegg (Verona, IT), and Darbo (Stans, A).

### 2.2 Preparation of probiotic yogurt

The different types of yogurts were collected on the day of their manufacturing. Briefly, pasteurized milk was subjected to ultrafiltration to achieve the desired fat and solid content, then to sterilization at 120°C for 30 sec and homogenization (APV, SPX Flow Technology, Crawley, United Kingdom). Milk was then incubated in a maturation tank at 38°C and inoculated with the starter culture (*Lactobacillus delbrueckii subs. bulgaricus* and *Streptococcus thermophilus)*. At the end of this process, when the desired pH was achieved, the clot was broken and the yogurt was cooled to stop the fermentation process. Fruit puree was then added and the yogurt was packed and stored at 4°C.

### 2.3 Lipid and protein composition of yogurt

Lipid fraction of plain yogurt (40 mg/mL) is composed of 69.14% saturated fats (palmitic acid [32%], stearic acid [10.8%], and myristic acid [10.7%]), 27.7% monounsaturated fats (oleic acid [24%] and palmitoleic acid [1.7%]), and 3.2% polyunsaturated fats (linoleic acid [2.7%] and linolenic acid [0.4%]). However, skim yogurt shows a very low concentration of fats (about 1 mg/mL) in comparison with plain yogurt, but maintains a similar percentage of saturated, monounsaturated, and polyunsaturated fatty acids. Of note, skim yogurt exhibits a protein content slightly higher (42 mg/mL) than plain yogurt (38 mg/mL). The reported data are provided by the manufacturer.

### 2.4 Preparation of yogurt aqueous extracts

For the preparation of the samples, aliquots of 0.25 mL of yogurt were extracted for 18 hr with 5 mL of distilled water at 4°C in an orbital shaker. The samples were then centrifuged at 20,000*g* for 20 min at 15°C. The supernatants were collected and filtrated through Whatman Chr. 1.

### 2.5 Determination of total phenolic content

Phenolic compounds were determined using the method described by Gülçin, (2012) with some modifications. Briefly, 1 mL of aqueous extract of yogurt, obtained as described above, was added to 1 mL of Folin‐Ciocalteau reagent diluted 1:2 with water. After 3 min, 2 mL of 10% Na_2_CO_3_ was added and the samples were incubated for 15 min at room temperature. At the end of this step, the absorbance was measured at 750 nm. A calibration curve was performed with gallic acid and the results were expressed as micrograms of gallic acid equivalents per 100 mL of sample (GAE).

### 2.6 Antioxidant activity of yogurt aqueous extracts estimated with the DPPH method

DPPH (1,1‐diphenyl‐2‐picrylhydrazyl) is a stable‐free radical whose reaction with antioxidant molecules can be followed as decrease in absorbance. The evaluation of the antioxidant activity was performed with the method described by Şengül et al. (2012) with some modifications. Yogurt extracts (0.02 mL) were diluted in 0.08 mL of water and then treated with 0.1 mL of 0.16 mmol/L DPPH dissolved in ethanol. The decrease in absorbance was measured spectrophotometrically at 517 nm. The percentage of antioxidant activity inhibition was calculated as:

%DPPHscavenging=(Abscontrol−Abssample)/(Abscontrol)×100

### 2.7 Antioxidant activity of yogurt aqueous extracts estimated with the ABTS method

ABTS (2, 2′‐azinobis(3‐ethylbenzothiazoline 6‐sulfonate)) can be oxidized to a stable radical cation (ABTS^•+^) which is decolorized after reaction with antioxidants. ABTS^•+^ was generated by reacting 7 mmol/L ABTS with 2.46 mmol/L potassium persulfate (final concentration) and the mixture was maintained at room temperature, in the dark, for 16 hr before use (Perna, Intaglietta, Simonetti, & Gambacorta, 2013). Then it was added to yogurt extracts to determine their antioxidant capacity. Briefly, 0.01 mL of water extracts of each type of yogurt was diluted in 0.09 mL of water and then treated with 0.1 mL of ABTS^•+^. The decrease in absorbance was measured at 415 nm with a plate reader. A calibration curve was performed with Trolox C and the results are expressed as Trolox C equivalent antioxidant capacity (TEAC).

### 2.8 Determination of lipid peroxidation

Lipid peroxidation was assessed as malondialdehyde (MDA) formation using the 2‐thiobarbituric acid assay as described by Yagi (1984) with modifications (Rigobello et al., 2008). Aliquots of 0.1 mL of yogurt were treated with 3.9 mL of 0.3 mol/L sulfuric acid and 0.5 mL of 10% phosphotungstic acid. After 10 min at 25°C, samples were centrifuged at 4,000*g* for 10 min. Pellets were resuspended in 2 mL of 0.3 mol/L sulfuric acid and 0.3 mL of 10% phosphotungstic acid and centrifuged again at the same speed. Then, samples were suspended with 1 mL of 0.67% thiobarbituric acid (TBA), 0.2 mL of 5% Nonidet, 0.04 mL of 1% BHT, and 2.8 mL of water and incubated for 1 hr at 95°C. After heat treatment, 3 ml of n‐butanol was added to the samples which were vigorously mixed and centrifuged at 6,000*g* for 5 min. The fluorescence of the upper phase was measured (Ex at 530 nm and Em at 540 nm). A calibration curve with 1,1,3,3‐tetraethoxypropane standard solution was used to quantitatively determine the concentration of thiobarbituric reactive substances (TBARS) in the samples.

### 2.9 Protein carbonyl content estimation

Protein oxidation was determined with the method described by Fenaille et al., (2006) with some modifications. Briefly, 2 mg proteins of the original yogurt were treated with 10 mmol/L 2,4‐dinitrophenylhydrazine (DNPH) dissolved in 2 mol/L HCl, for 30 min in the dark. Proteins were then precipitated with 10% TCA and centrifuged for 5 min at 10,000*g*. To remove unreacted DNPH, the obtained pellets were washed three times with 1 mL of ethanol/ethyl acetate (50:50), and dissolved with 8 mol/L guanidine (pH 2.3). Absorbance was estimated at 370 nm and protein concentration was determined by the Bradford assay, (Bradford, 1976) using 0.01 mL of samples dissolved with guanidine.

### 2.10 Statistical analysis

Each analysis of the samples was performed once a week from the production to 2 weeks after the shelf life. All the experiments reported are the mean with respective SD of, at least, four experiments. The statistical analysis of variance (ANOVA) was performed using Tukey test with INSTAT 3.3 (GraphPad) software.

## 3 RESULTS AND DISCUSSION

### 3.1 Total phenolic content of different types of yogurt

**Figure 1:**
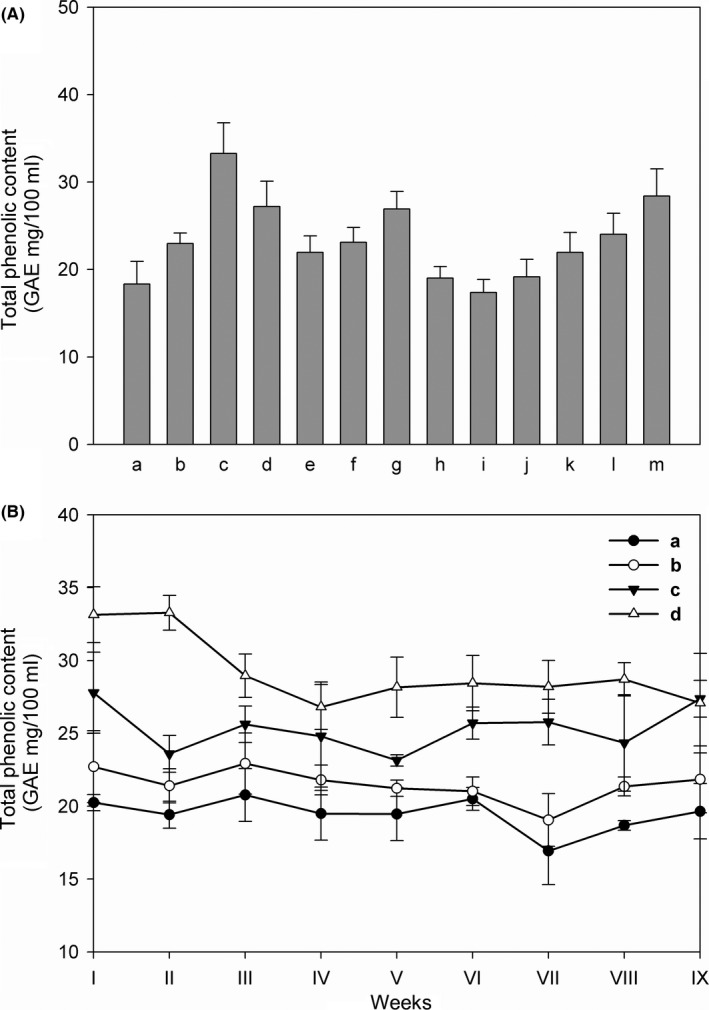
Determination of total phenolic content (TPC) in different types of yogurt. (A) yogurt aqueous extracts (1 mL) were tested and TPC was determined by the Folin‐Ciocalteau method. (a) plain yogurt; (b) skim yogurt; (c) plain berries yogurt; (d) skim berries yogurt; (e) plain strawberries yogurt; (f) plain blueberries yogurt; (g) plain cherry yogurt; (h) plain pineapple and orange yogurt; (i) plain plum yogurt; (j) plain apricot yogurt; (k) skim apple yogurt; (l) skim banana yogurt; m: skim peach yogurt. (B) Determination of total phenolic content (TPC) of (a–d), during the shelf life. The experiments were performed once a week for 9 weeks. Total phenolic content was expressed as mg of gallic acid equivalents (GAE) per 100 mL of yogurt

The concentration of total phenolic groups (TPC) in yogurt samples treated with different fruit purees was analyzed. Figure 1A reports the TPC content at the beginning of the storage, estimated as micrograms of gallic acid equivalents (GAE) per 100 mL of sample. The addition of fruit puree determined an increase in total phenolic groups, particularly when berries (c, d), cherry (g), and peach (m) purees were present. Tests were repeated during the shelf life by choosing two types of yogurt, plain and skim with and without berries, and the results are reported in Figure 1B. The duration of storage determined a slight decline of TPC mostly in skim yogurt treated with puree of berries (d), whereas for plain (a), skim (b), and plain with berries (c) yogurts the content did not change. Therefore, data analysis shows that during the shelf life, the products are not significantly altered regarding their TPC.

### 3.2 Antioxidant capacity of different types of yogurts

#### 3.2.1 DPPH radical scavenging during the shelf life of yogurts

**Figure 2:**
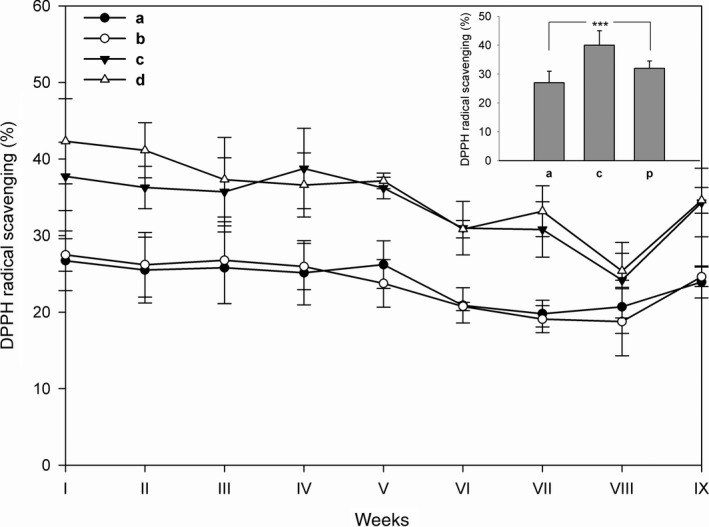
Determination of the antioxidant activity with DPPH in various types of yogurt during the shelf life. Aliquots (20 μL) of yogurt extracts were treated with 0.08 mmol/L DPPH and the decrease in absorbance was estimated at 517 nm. The analysis was repeated once a week for 9 weeks. The activity was expressed as % of DPPH radical scavenging. (a) plain yogurt; (b) skim yogurt; (c) plain berries yogurt, and (d) skim berries yogurt. The inset reports the antioxidant activity of plain yogurt (a), yogurt added with berries puree (c), and berries puree alone (p) treated in the same conditions. ***p < 0.001

Highly reactive free radicals and other reactive oxygen species generated from a wide variety of sources occur in biological systems. However, these species are efficiently recognized by antioxidants. DPPH is a relatively stable organic‐free radical which has been widely used to test the free radical scavenging ability of various samples containing antioxidant molecules. The method is based on the reduction in alcoholic DPPH solutions in the presence of an antioxidant, as DPPH solutions show a deep violet color with a strong absorption band at 517 nm which decreases in the presence of antioxidant molecules. The resulting bleaching of DPPH is stoichiometric with the antioxidant molecules examined and the estimation of the consumed DPPH radicals is a measure of the radical scavenging activity (Kulisic, Radonic, Katalinic, & Milos, 2004). The results reported in Figure 2 refer to plain (a), skim (b), plain berries (c), and skim berries (d) yogurt extracts. The DPPH scavenging activity of yogurt samples increased in the presence of berries puree, however, berries puree alone does not show the same percentage of scavenging activity (inset of Figure 2, column p) respect to the control of plain berries yogurt. In addition, the antioxidant power decreased during the shelf life until the eighth week and after increased to values comparable to those observed at the beginning. As discussed in the next paragraph (3.2.2), this biphasic behavior is probably due to the late formation of peptides with antioxidant properties, deriving from the fragmentation of milk proteins (Perna et al., 2013; Dziuba & Dziuba, 2014).

#### 3.2.2 Measurement of ABTS radical scavenging activity

As a complement to the previous estimation, the antioxidant capacity of aqueous extracts of yogurt samples using the ABTS test, was also determined. First of all, a calibration standard curve with Trolox C was prepared and, the data are expressed as equivalents of transformed Trolox C (TEAC). During the shelf life significant differences were observed, mainly for plain and skim yogurt, compared with the same yogurts with berries. As reported in Table 1, in the first week, the antioxidant capacity ranges from 835 μmol/L Trolox equivalents for plain yogurt to 1,853 μmol/L Trolox equivalents for plain berries yogurt and from 1,044 μmol/L Trolox equivalents for skim yogurt to 1,925 μmol/L Trolox equivalents for skim berries yogurt, once again highlighting that the presence of polyphenols is relevant for the antioxidant power. In addition, during the shelf life, in the third and fifth week, we can observe a marked decrease in antioxidant capacity, reaching 50%, for plain and skim yogurts in the fifth week. However, in the seventh week, the antioxidant capacity increases in accordance with the antioxidant activity pattern seen with the DPPH method (see above). Again, like previously observed, this behavior seems to depend on the generation of bioactive peptides deriving from the proteolytic cleavage of caseins and other milk proteins endowed with antioxidant properties (Pihlanto, 2006; Perna et al., 2013; Dziuba & Dziuba, 2014). Of note, the proteolytic activity can determine the release of antioxidants naturally present in the yogurt samples and entrapped in globular proteins such as caseins (Trigueros, Wojdyło, & Sendra, 2014).

**Table 1:** ABTS radical scavenging activity of water‐soluble yogurt extracts

Type of yogurt	Trolox equivalent antioxidant capacity (μmol/L Trolox/mL of yogurt)			
	Weeks			
	I	III	V	VII
A	835.2 ± 24.2	720.9 ± 105.3	409.2 ± 74.5	941.2 ± 84.3
B	1044.3 ± 64.3	947.7 ± 109.6	441.2 ± 63.1	912.1 ± 80.7
C	1853.8 ± 104.1	1372.0 ± 198.3	1457.1 ± 251.3	1756.9 ± 341.9
D	1925.2 ± 208.6	1582.2 ± 240.3	1568.3 ± 170.3	1811.7 ± 298.1

Aliquots (10 μL) of aqueous yogurt extracts were treated with 0.04 mmol/L ABTS, and the decrease in absorbance was estimated at 415 nm with a plate reader. The values obtained were compared with a standard curve of Trolox C and expressed as TEAC or Trolox equivalent antioxidant activity (μmol/L Trolox C/mL of yogurt). (A) plain yogurt; (B) skim yogurt; (C) plain berries yogurt; (D) skim berries yogurt.

#### 3.2.3 Estimation of lipid peroxidation in basal or stimulated conditions

**Figure 3:**
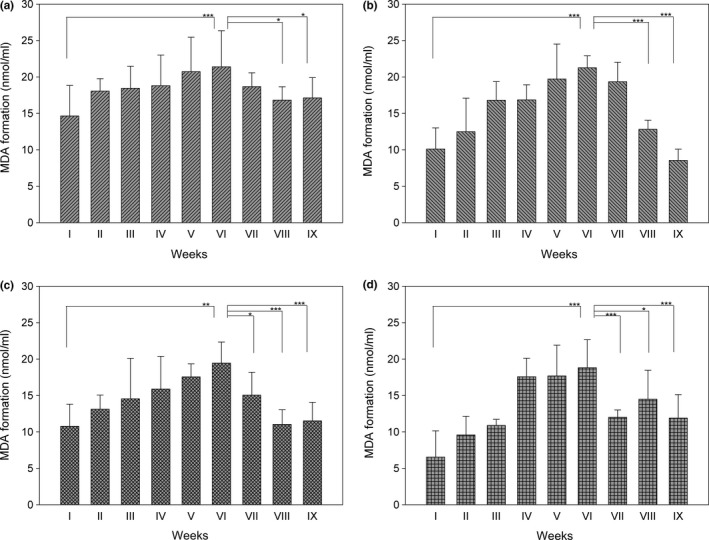
Estimation of malondialdehyde during the shelf life of yogurt samples. Lipid peroxidation was evaluated during yogurt storage (weeks). Aliquots of 0.1 mL of yogurt were subjected to MDA determination as described in Experimental. Fluorometric analysis was performed after extraction of malondialdehyde with *n*‐butanol (λ_Ex_‐ 530 nm, λ_Em_‐ 540 nm). For the quantification of MDA, a standard curve with 1,1,3,3‐tetraethoxypropane was performed. (a) plain yogurt; (b) skim yogurt; (c) plain berries yogurt; (d) skim berries yogurt. *p <* *0.05; **p <* *0.01; ***p <* *0.001

**Figure 4:**
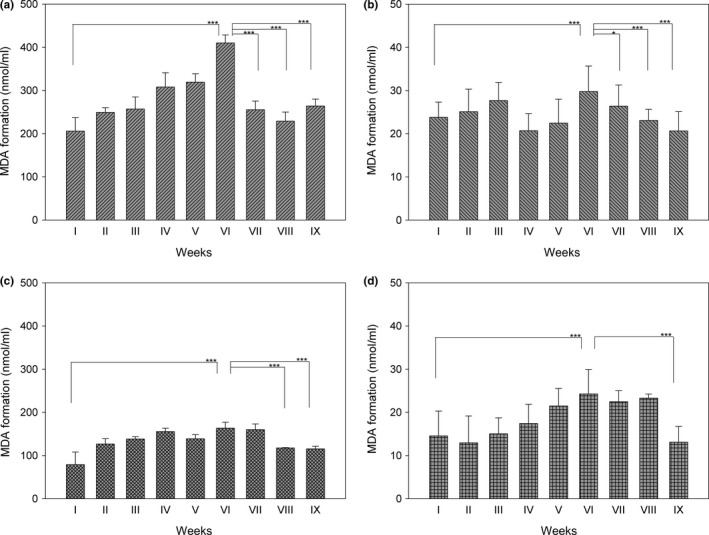
Estimation of malondialdehyde after treatment of yogurt samples with cumene hydroperoxide/hemin. Lipid peroxidation was evaluated during yogurt sample storage for a period of 9 weeks. Briefly, aliquots of 0.1 mL of yogurt were treated for 30 min with 25 mmol/L cumene hydroperoxide/0.05 mmol/L hemin, and then subjected to MDA determination as described in Experimental. After extraction of malondialdehyde with butanol, fluorometric analysis was performed (λ_Ex_‐ 530 nm; λ_Em_‐ 540 nm). (a) plain yogurt; (b) skim yogurt; (c) plain berries yogurt; (d) skim berries yogurt. For quantification of MDA, a calibration curve with 1,1,3,3‐tetraethoxypropane as reference standard was used to determine the concentrations of TBARS in the samples. *p <* *0.05; **p <* *0.01; ***p <* *0.001

To assess the spontaneous lipoperoxidation of yogurt occurring during the shelf life, a test based on malondialdehyde (MDA) production was carried out. Yogurts were collected at the end of the packaging and stored at 4°C over the period of the shelf life. The analyses were carried out at the indicated weeks until the end of the established shelf life (50 days) and, in addition, up to 20 days after the expiration date. As reported in Figure 3, the assays were carried out for 9 weeks for the plain (panel A), skim (panel B), plain berries (panel C), and skim berries (panel D) yogurts, respectively, using 0.1 mL of sample. First of all, skim and skim berries yogurts show lower levels of MDA, especially in stimulated conditions by addition of cumene hydroperoxide/hemin (Figure 4, see below), due to a minor content of lipids. In addition, we observed a slight increase in lipoperoxidation particularly in the fifth or sixth week especially in plain yogurt. However, the levels of lipid peroxidation were relatively low reaching a maximum of about 21 nmoles/mL (Figure 3a and c). In the presence of berries, MDA level was even lower both in plain and skim yogurt samples. Of note, at longer times (VII to IX weeks) a decrease in MDA formation, especially in plain and plain with berries yogurts, was observed.

However, when lipid peroxidation was stimulated by addition of cumene hydroperoxide/hemin, a strong increase in MDA formation was observed. As apparent in Figure 4, a rapid increase in MDA production, especially for plain yogurt (Figure 4a) compared to skim yogurt (Figure 4b), was detected, in agreement with the lower fat content present in the skim yogurt. In addition, during the shelf life, a constant increase in MDA formation until the sixth week was observed. This behavior was found both in the presence and in the absence of berries. However, in the presence of berries a lower MDA production with respect to plain yogurt alone is apparent by comparing Figure 4a (400 nmoles/mL at the sixth week) with Figure 4c (150 nmoles/mL at the sixth week). Similarly, the presence of berries determines a decrease in lipoperoxidation of skim yogurt compared to skim yogurt without berries, but MDA values result far lower (Figure 4b and d). Notably, MDA formation both in basal condition and in the presence of cumene hydroperoxide/hemin was also estimated in a home‐made yogurt, however, very high values of lipid peroxidation already at 48 hr after production were found (data not shown), indicating that controlled conditions of manufacturing ensure a better product. According to Serra, Trujillo, Pereda, Guamis, & Ferragut (2008) the low pH, the temperature of maintenance of the product during the shelf life and the material of packaging generally with low permeability to oxygen, make lipid peroxidation a secondary problem. However, the quality of milk used to obtain the yogurt and some fortifications, for example, with iron, determine a dramatic increase in lipid peroxidation (Hekmat & McMahon, 1997). Therefore, the results of Figure 4 clearly show that when MDA was stimulated in the presence of cumene hydroperoxide/hemin, a net increase in MDA formation until at least 7 weeks takes place, indicating a potential susceptibility of yogurt to peroxidative processes.

As apparent in Figures 3 and 4, after 6 weeks of shelf life, a decrease in MDA formation was observed in all the samples. This occurrence is probably associated with the activity of bacteria present in the yogurt and producing bioactive peptides. A preliminary analysis of these peptides indicates that some of them are endowed with antioxidant properties. We have performed some preliminary studies on the proteolytic process, and, using the o‐phthalaldehyde (OPA) based fluorescent assay, we noticed that there was an effective increase in peptides formation during the shelf life, strictly dependent on the temperature of conservation of the product (data not shown). Bioactive peptides, in addition to other functions, exert also an antioxidant role behaving both as free radical scavengers or chelators of transition metal ions (Sarmadi & Ismail, 2010). Usually, they are inactive when present in the sequence of the original proteins but they gain activity once released by enzymatic hydrolysis of the parent protein (Power et al., 2013; Pihlanto, 2006). In addition, they are also able to stimulate the expression of genes coding for antioxidant enzymes such as heme oxygenase, glutathione peroxidase, catalase, and superoxide dismutase (Sarmadi & Ismail, 2010).

### 3.3 Estimation of protein carbonyl group formation

**Figure 5:**
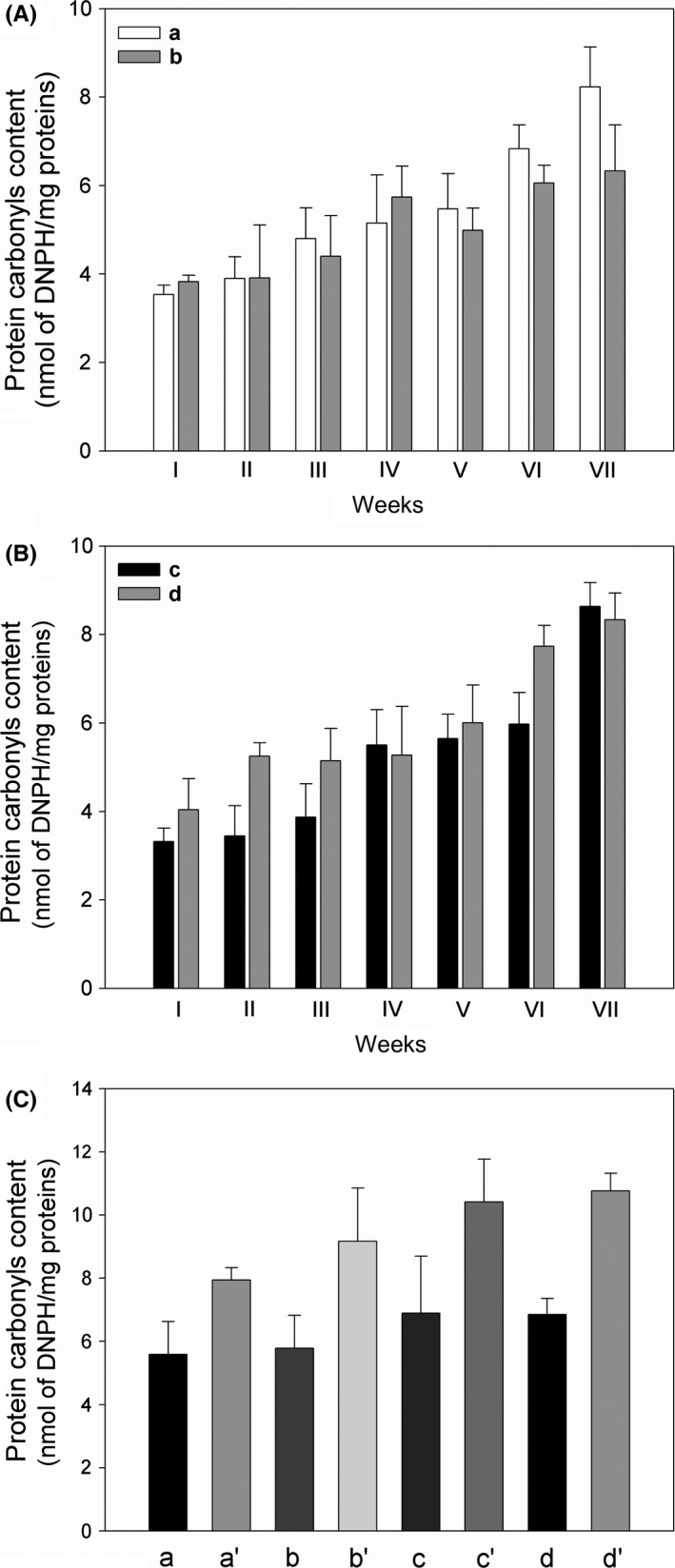
Protein carbonyls content during the shelf life. Aliquots of yogurt (2 mg) were treated with 20 mmol/L DNPH for 30 min in the dark, then precipitated with 10% TCA and washed pellets were resuspended with 8 M guanidine (pH 2.3). The content of carbonyl was estimated spectrophotometrically at 370 nm. (A) plain (a) and skim (b) yogurt; (B) plain berries (c), and skim berries (d) yogurt; (C) carbonyls content in the absence (a), (b), (c), (d) and in the presence (a’), (b’), (c’) (d’) of 25 mmol/L cumene hydroperoxide/0.05 mmol/L hemin, at the fifth week of shelf life

During the fermentation process of yogurt, proteins are partially hydrolyzed into peptides and free amino acids (Germani et al., 2014). The yogurt samples (plain, skim, plain berries, and skim berries) were also tested for protein carbonyl formation during the shelf life, at the same weeks in which lipid peroxidation was measured. The amount of protein carbonyl groups (Figure 5) was very similar in the absence (Figure 5A, a, b) or in the presence (Figure 5B, c, d) of berries, indicating that berries were unable to prevent protein oxidation and in addition, skim yogurt, which has a higher protein content, showed similar values of carbonyl content in comparison to plain yogurt. This result is quite different from that obtained for MDA formation (Figures 3 and 4) indicating that the antioxidants of yogurt are more prone to protect against lipid peroxidation, but seem scarcely able to influence protein oxidation. During the shelf life of 45 days, the proteolytic activity of bacteria in yogurt proceeds (Germani et al., 2014) and determines a fragmentation and an oxidation particularly in plain yogurt. Protein oxidation is higher compared with skim product at the seventh week (Figure 5A), but this does not occur for the samples (plain or skim) containing puree of berries (Figure 5B). In addition, carbonyl content after the incubation of yogurts with cumene hydroperoxide/hemin was estimated (Figure 5C). Also in this case, the presence of berries do not reduce the extent of protein oxidation.

## 4 CONCLUSION

The antioxidant activity of yogurt samples depends on the endogenous antioxidants, on added antioxidants contained in fruit puree and on the formation of bioactive peptides. The antioxidant properties of bioactive peptides depend on their specific amino acid composition and Tyr, Cys, and His residues are particularly relevant. In addition, some peptides may also be able to exert a marked synergistic effect with phenolic antioxidants (Saito et al., 2003). The production of endogenous antioxidant peptides, and the incidence of added antioxidants present in the puree undoubtedly constitute a good protection against a lipid peroxidation, and therefore reduce the peroxidizability of yogurt. On the contrary, the oxidation of the proteins is not prevented by the antioxidants present in yogurt. In addition, DPPH and ABTS assays show that the total antioxidant capacity slowly decreases during the shelf life. However, after a relatively long storage an increase in oxidant species scavenging capability was observed and attributed to the production of bioactive peptides. Probably, hydrolyzed proteins may also act as “sacrificial” antioxidants (Halliwell & Gutteridge, 2015) and this might explain the observed different behavior between lipid and protein oxidation toward oxidants. In conclusion, the specific prevention of lipid peroxidation products formation by endogenous and added antioxidants improves the nutritional quality of yogurt.

## CONFLICT OF INTEREST

Authors declare no conflict of interest.
